# Delayed intensive care unit admission from the emergency department: impact on patient outcomes. A retrospective study

**DOI:** 10.5935/0103-507X.20210014

**Published:** 2021

**Authors:** Waleed Tharwat Aletreby, Peter G. Brindley, Ahmed Naji Balshi, Basim Mohammed Huwait, Abdulrahman Mishaal Alharthy, Ahmed Fouad Madi, Omar Elsayed Ramadan, Alfateh Sayed Nasr Noor, Wasim S. Alzayer, Mohammed A. Alodat, Hend Mohammed Hamido, Shahzad Ahmed Mumtaz, Abdullah Balahmar, Papas Vasillios, Huda Mhawish, Dimitrios Karakitsos

**Affiliations:** 1 Department of Critical Care, King Saud Medical City - Riyadh, Saudi Arabia.; 2 University of Alberta - Alberta, Canada.; 3 Department of Anesthesia, Faculty of Medicine, Tanta University - Tanta, Egypt.; 4 Department of Anesthesia, Faculty of Medicine, Ain-Shams University - Cairo, Egypt.; 5 Department of Obstetrics and Gynecology, King Saud Medical City - Riyadh, Saudi Arabia.; 6 Department of Critical Care, Keck School of Medicine, University of Southern California - Los Angeles, California, United States.

**Keywords:** Emergency service, hospital, Hospital mortality, Length of stay, Risk factors, Intensive care units, Serviço hospitalar de emergência, Mortalidade hospitalar, Tempo de internação, Fatores de risco, Unidades de terapia intensiva

## Abstract

**Objective:**

To study the impact of delayed admission by more than 4 hours on the outcomes of critically ill patients.

**Methods:**

This was a retrospective observational study in which adult patients admitted directly from the emergency department to the intensive care unit were divided into two groups: Timely Admission if they were admitted within 4 hours and Delayed Admission if admission was delayed for more than 4 hours. Intensive care unit length of stay and hospital/intensive care unit mortality were compared between the groups. Propensity score matching was performed to correct for imbalances. Logistic regression analysis was used to explore delayed admission as an independent risk factor for intensive care unit mortality.

**Results:**

During the study period, 1,887 patients were admitted directly from the emergency department to the intensive care unit, with 42% being delayed admissions. Delayed patients had significantly longer intensive care unit lengths of stay and higher intensive care unit and hospital mortality. These results were persistent after propensity score matching of the groups. Delayed admission was an independent risk factor for intensive care unit mortality (OR = 2.6; 95%CI 1.9 - 3.5; p < 0.001). The association of delay and intensive care unit mortality emerged after a delay of 2 hours and was highest after a delay of 4 hours.

**Conclusion:**

Delayed admission to the intensive care unit from the emergency department is an independent risk factor for intensive care unit mortality, with the strongest association being after a delay of 4 hours.

## Abstract (pt)

**Objetivo:**

Estudar o impacto do retardo na admissão à unidade de terapia intensiva em mais do que 4 horas nos desfechos de pacientes críticos.

**Métodos:**

Este foi um estudo observacional retrospectivo, no qual pacientes adultos admitidos diretamente do pronto-socorro para a unidade de terapia intensiva foram divididos em dois grupos: Tempo Adequado, se admitidos dentro de 4 horas, e Admissão Retardada, nos casos em que a admissão demorou mais do que 4 horas para ocorrer. Compararam-se, entre os grupos, o tempo de permanência na unidade de terapia intensiva e a taxa de mortalidade na unidade de terapia intensiva e no hospital. Foi realizado pareamento por escore de propensão para correção de desequilíbrios. Utilizou-se uma análise de regressão logística para explorar retardo da admissão como fator independente de risco para mortalidade na unidade de terapia intensiva.

**Resultados:**

Durante o período do estudo, 1.887 pacientes foram admitidos diretamente do pronto-socorro para a unidade de terapia intensiva, sendo que 42% dessas admissões foram retardadas. Os pacientes com retardo tiveram permanências na unidade de terapia intensiva significantemente mais longas e maior mortalidade na unidade de terapia intensiva e no hospital. Esses resultados persistiram após pareamento dos grupos por escore de propensão. O retardo da admissão foi fator independente de risco para mortalidade na unidade de terapia intensiva (RC = 2,6; IC95% 1,9 - 3,5; p < 0,001). A associação de retardo e mortalidade na unidade de terapia intensiva surgiu após período de retardo de 2 horas e foi mais alta após período de retardo de 4 horas.

**Conclusão:**

O retardo da admissão do pronto-socorro para a unidade de terapia intensiva é fator de risco independente para mortalidade na unidade de terapia intensiva, sendo a associação mais forte após retardo de 4 horas.

## INTRODUCTION

Delayed admission to the intensive care unit (ICU) from the emergency department (ED) could be due to myriad reasons. These include the growing need for ICU admission because ED patients are increasingly elderly, frail and complex.^(1-3)^ There is also competing pressure for ICU beds from wards and operating theaters.^(4,5)^ The concern is that delayed ICU admission translates into delays in time-sensitive care. Delays in care could be specific, such as the need for immediate thrombolysis, early resuscitation,^(1)^ the implementation of sepsis protocols,^(2)^ and emergent needs for revascularization, fluid resuscitation, and antibiotics.^(6)^ More generally, expeditious ICU admission could also mean earlier attention from intensivists, more one-on-one nursing, and closer monitoring.^(7)^ Regardless, the problem appears to be worsening,^(8)^ as reflected by reports of up to 75% of ICU admissions being delayed over 4 hours and patients being boarded in the ED for over 3 days.^(2)^

Previous studies have explored the impact of delayed ICU admission from the ED, but the results have been conflicting. Some found no association,^(7,9,10)^ whereas others did find an association but could not establish at what point the delay became clinically detrimental.^(1-3,11)^ This study intended to explore these two questions at our institution. We chose a 4-hour cutoff of admission per our institutional policy.

## METHODS

This was a retrospective observational study from the ICU of King Saud Medical City, Riyadh, Saudi Arabia. It received ethics approval from our institutional review board with a waiver of informed consent (H1RI-08-Oct19-02) and utilized the STROBE checklist of minimal reporting in observational studies.^(12)^ King Saud Medical City is the largest Ministry of Health hospital in the Kingdom, with 1,200 inpatient beds, of which 125 are ICU beds (as defined by the ability to administer mechanical ventilation and inotropes, one-on-one nursing, and a specialist/consultant intensivist as the most responsible physician).

The hospital provides 24/7 laboratory, radiology, and surgical services. The ICU is divided into subunits (medical, surgical, respiratory, trauma, and neurocritical). The mean ICU monthly admission rate was 270 patients, the average mortality was 15%, the average bed occupancy was 95%, and the average length of stay (LOS) was 10 days. Our hospital lacks a step-down unit. Intensive care unit referrals from the ED are reviewed by an intensive care consultant/specialist, and decisions regarding admission and discharge involve a registered critical care nurse and a respiratory therapist. If an ICU bed is not available within 60 minutes, the ICU team comanages the patient with the primary team in the ED until a bed becomes available. ICU admission prioritization is up to the attending consultants on duty and based on their evaluation of the clinical condition, prognosis, and bed availability. It is a key performance indicator of the ICU to transfer accepted patients from the ED within 4 hours.

We retrospectively reviewed the records of ICU referrals from the ED between January 1^st^, 2018, and December 31^st^, 2019. We excluded all patients deemed not appropriate for ICU admission. We reviewed all patients accepted for ICU admission except for those aged < 18 years, pregnant, who died in the ED before admission, left the ED before admission (i.e., left against medical advice or were transferred to other hospitals) or were admitted to the ICU after surgical procedures; however, patients admitted under the care of surgery without a surgical intervention prior to ICU admission were included.

For all included patients, we recorded their age, sex, diagnosis and general diagnostic category (medical, surgical, and trauma), mechanical ventilation status, need for vasopressors, need for continuous renal replacement therapy (CRRT), insertion of a central venous line, measures of severity such as Acute Physiology and Chronic Health Evaluation (APACHE) 4, Sequential Organ Failure Assessment (SOFA) score, Modified Early Warning Sore (MEWS), and sepsis status. We recorded the time between referral and physical transfer to the ICU (in minutes). Finally, from the patients' medical records, we recorded the ICU LOS, ICU mortality and in-hospital mortality. We differentiated patients into two groups: Timely, if admission to the ICU occurred within 240 minutes (i.e., 4 hours), and a Delayed Group if it was > 240 minutes. Several a priori set subgroup analyses were performed: age (above or below the cohort median), gender, mechanical ventilation, vasopressors, APACHE IV (above or below the cohort median), and the presence or absence of sepsis. We utilized the sepsis definition by The Third International Consensus Definitions for Sepsis and Septic Shock (Sepsis-3).^(13)^

The primary outcome of the study was the independent association of a delayed admission and ICU mortality. Secondary outcomes were combined ICU and in-hospital mortality and the average ICU LOS. We also studied the length of delay after which a significant impact on ICU mortality appeared. We chose ICU mortality, as it is one of the quality indicators of ICU effectiveness,^(14)^ with the advantages of being a patient-important outcome, less prone to biases, and easier to communicate to readers. Furthermore, the regulatory authorities mandate proof of improved short-term mortality before the approval of new therapies in critical care.^(15)^

### Statistical methods

To explore the association between delayed admission and ICU outcomes, we evaluated several variables in a univariate logistic regression (LR) model. Variables with p-values < 0.1 or those judged to have a clinically significant impact on ICU mortality were subsequently entered into a multivariate LR model, and the results were reported as odds ratios (OR) with corresponding 95% confidence intervals (95%CI). The goodness of fit of the model was evaluated by the Hosmer-Lemeshow test (considered well fitted with p > 0.05) and the area under the curve. The absence of multicollinearity of the independent variables was evaluated by the Variable Inflation Factor (VIF) after removing any variable with VIF ≥ 5.^(16)^ The linearity of the independent variables and log odds was explored by the Box-Tidwell test, satisfying the assumption of a p-value > 0.05.^(17)^ As a sensitivity measure, we performed a stepwise inclusion of significant variables in the LR model to evaluate their impact on the crude OR of ICU mortality regressed on delayed admission, and then we evaluated all relevant estimates.^(18)^ The final multivariate LR model was repeated several times with the same variables, changing the delay definition each time from 1 hour to 10 hours.

Separately, we performed propensity score matching (PSM) of patients admitted after 240 minutes using those admitted within 240 minutes as controls. Groups were matched 1:1 by age, sex, mechanical ventilation, vasopressors, CRRT, sepsis, SOFA and APACHE IV scores, with a caliber of 0.03. Group comparisons and LR were repeated for the matched groups.

Evaluation of the confounding effect of different strata used in the subgroups was performed using the Mantel Haenszel method, evaluated by Tarone's test of homogeneity of OR,^(19)^ considering OR across strata to be homogeneous with a p-value > 0.1.

Continuous variables are presented as the mean ± standard deviation (SD) and were compared between groups with Student's t-test or the Mann-Whitney test as appropriate. Categorical variables are presented as numbers (%) and were compared with chi-squared or Fisher's exact test as appropriate. All statistical tests were two-tailed and considered significant if the p-value < 0.05, with no correction for multiple testing.

A minimum sample size of 1,452 (at least 726 in each group) was calculated to significantly detect a reduction of 5% in ICU mortality (assuming a 15% rate in the control group) with 80% power and a type I error of 5%. Based on our historical data where almost half of our admissions were from the ED, we postulated that recorded data over 2 years would be sufficient to power our study.

Statistical tests were performed using a commercially available software package (StataCorp. 2019. Stata Statistical Software: Release 16. College Station, TX: StataCorp LLC.).

## RESULTS

### Groups' comparison (unmatched and matched)

In 2018 and 2019, there were 4,147 referrals to the ICU from the ED; 2,260 were excluded, meaning 1,887 were included in the study (Figure 1). Missing data were minimal (the highest was 159; 8.4% without a MEWS value). Missing values were imputed by multiple imputation (Table 1S - Appendix). Intensive care unit and hospital outcomes were 100% complete for the study cohort. Among the included patients, 1,093 (58%) were admitted within 240 minutes with an average time to admission of 158 ± 81.1 minutes, whereas 794 (42%) were delayed, with an average time to admission of 625.2 ± 485.4 minutes.

**Figure 1:**
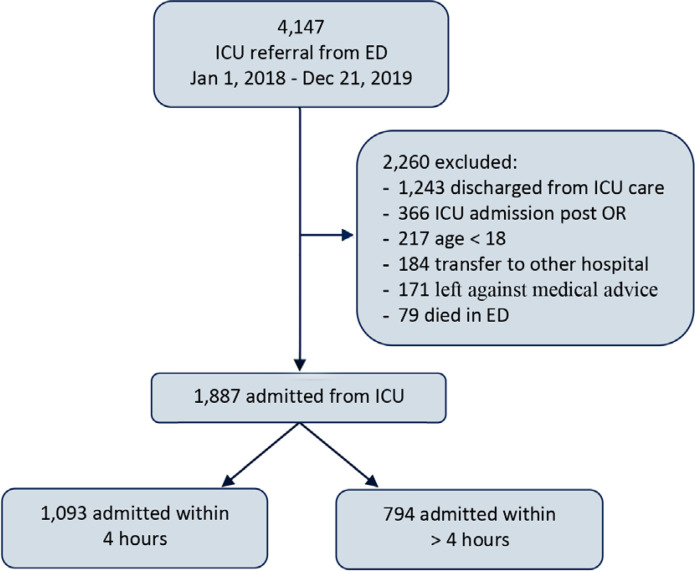
Patients' flow diagram.

ICU - intensive care unit; ED - emergency department; OR - operative room.

Group comparisons showed demographic and clinical differences between the groups. Patients admitted within 4 hours were significantly younger, with higher severity scores (APACHE IV and SOFA), but less frequently required continuous CRRT. They were also more often surgical patients and less often medical, while trauma cases were distributed evenly between the groups (Table 1). The top five diagnoses in each category are presented in table 2S (Appendix).

**Table 1:** Demographic and clinical characteristics at the emergency department

	Delayed (n = 794)	Within time (n = 1,093)	p value
Males	574 (72.3)	784 (71.7)	0.8
Age	49.9 ± 19.9	46.8 ± 19.3	< 0.001
Diagnosis			
Medical	558 (70.3)	675 (61.8)	< 0.001
Surgical	110 (13.9)	246 (22.5)	< 0.001
Trauma	126 (15.8)	172 (15.7)	0.99
MV	317 (39.9)	463 (42.4)	0.3
Central venous line	577 (72.7)	805 (73.7)	0.6
Vasopressors	462 (58.2)	664 (60.8)	0.3
CRRT	191 (24.1)	142 (13)	< 0.001
Sepsis	147 (18.5)	92 (8)	< 0.001
APACHE IV	65.9 ± 2.8	66.5 ± 3.1	< 0.001
SOFA	7.1 ± 2.9	7.7 ± 3	< 0.001
MEWS	2.4 ± 2	2.6 ± 2.3	0.06
Time to admission (minute)	625.2 ± 485.4	158 ± 81.1	< 0.001

MV - mechanical ventilation; CRRT - continuous renal replacement therapy; APACHE - Acute Physiology and Chronic Health Evaluation; SOFA - Sequential Organ failure Assessment; MEWS - Modified Early Warning Score. Results expressed as n (%) or mean ± standard deviation.

The Timely Admission Group had a significantly shorter ICU LOS (9.4 ± 11.3 days) than the Delayed Admission Group (15.2 ± 17.1 days, p < 0.001). The Timely Admission Group also had a significantly lower ICU mortality (10.4% *versus* 28.8%; p < 0.001) and in-hospital mortality (14.8% *versus* 35.1%; p < 0.001) (Table 2).

**Table 2:** Outcomes of delayed and within-time admissions

	Delayed (n = 794)	Within time (n = 1,093)	p value
ICU length of stay	15.2 ± 17.1	9.4 ± 11.3	< 0.001
ICU mortality	229 (28.8)	114 (10.4)	< 0.001
Hospital mortality	279 (35.1)	162 (14.8)	< 0.001

ICU - intensive care unit. Results expressed as mean ± standard deviation or n (%).

Propensity score matching balanced the groups (except for sepsis distribution) with 794 observations each (Table 3S, Figure 1S - Appendix). The matched timely group had a shorter ICU LOS (12 ± 14.1 *versus* 15.2 ± 17.1; p < 0.001), lower ICU mortality (13.1% *versus* 28.8%; p < 0.001) and lower hospital mortality (19% *versus* 35.1%, p < 0.001) (Table 4S - Appendix).

### Association of delay and intensive care unit mortality

The multivariate LR model included demographic and clinical characteristics with a p-value < 0.1 in a univariate LR analysis (age, mechanical ventilation, vasopressors, central venous line, diagnostic category, APACHE IV score, sepsis, time to admission, ICU LOS, and delayed admission), in addition to variables judged to clinically influence the ICU outcomes (CRRT, SOFA) regardless of their p-value (Table 3). The model was well fitted with a Hosmer Lemeshow p-value = 0.8 and an area under the curve of 0.82 (95%CI 0.8 - 0.84). The assumptions of an LR were satisfied (Tables 5S and 6S - Appendix).

**Table 3:** Risk factors for intensive care unit mortality

Variable	Univariate model			Multivariate model		
	OR	95%CI	p value	OR	95%CI	p value
Age	1.02	1.01 - 1.03	< 0.001	1.01	1.001 - 1.02	< 0.001
Sex	1.03	0.8 - 1.3	0.8			
MV	1.6	1.3 - 2.05	< 0.001	1.7	1.3 - 2.3	< 0.001
CRRT	1.09	0.8 - 1.5	0.6	2.4	1.5 - 4	< 0.001
Vasopressors	0.7	0.6 - 0.9	0.01	0.4	0.16 - 1.05	0.06
Central venous line	0.6	0.5 - 0.8	0.001	0.9	0.6 - 1.4	0.6
Diagnosis						
Medical	Referência					
Surgical	0.9	0.7 - 1.2	0.6	1.1	0.9 - 1.3	0.4
Trauma	0.6	0.4 - 0.8	0.005	0.9	0.6 - 1.5	0.7
APACHE IV	1.06	1.02 - 1.1	0.003	1.04	0.99 - 1.1	0.1
SOFA	1.02	0.98 - 1.05	0.4	1.2	1.1 - 1.4	< 0.001
MEWS	1.03	0.97 - 1.1	0.3			
Sepsis	4.3	3.3 - 5.8	< 0.001	10.2	6.7 - 15.5	< 0.001
Time to admission	1.001	1.0007 - 1.0012	< 0.001	1.0006	1.0002 -1.001	0.001
ICU length of stay	1.03	1.02 - 1.04	< 0.001	1.02	1.01 - 1.03	< 0.001
Delay Admission	3.5	2.7 - 4.5	< 0.001	2.6	1.9 - 3.5	< 0.001

OR - odds ratio; 95%CI - 95% of confidence interval; MV - mechanical ventilation; CRRT - continuous renal replacement therapy; APACHE - Acute Physiology and Chronic Health Evaluation; SOFA - Sequential Organ failure Assessment; MEWS - Modified Early Warning Score; ICU - intensive care unit.

Delayed admission (i.e., more than 4 hours from the ED to the ICU) was independently associated with an increased likelihood of ICU mortality (OR = 2.6; 95%CI 1.9 - 3.5; p < 0.001). Other variables also associated with an increased likelihood of ICU mortality were older age, a need for mechanical ventilation, dialysis performed in the ED, a higher SOFA score, sepsis, a longer time in the ED, and a longer ICU LOS.

We assessed the sensitivity of our results by a statistical method^(18)^ that describes all possible estimates and changes in estimates. Figure 2 shows that all possible estimates are above the horizontal line of the null value (OR = 1) and to the left (less than) of the vertical line representing a p-value of 0.05. The change in estimates is shown in figure 3, in which after adding potential confounders to the crude estimate in a stepwise fashion (starting with the largest effect), the OR of delayed admission on the ICU outcome remained significant and overlapped with the crude OR, reinforcing the robustness of the LR model's results.

**Figure 2:**
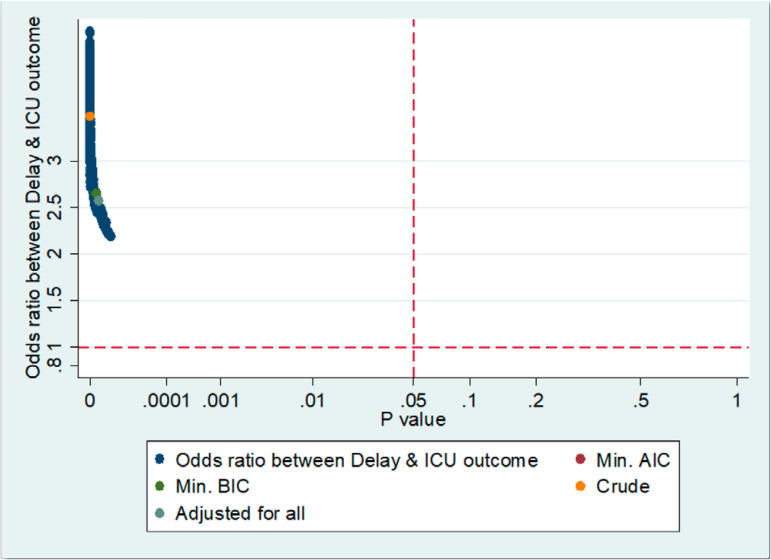
Estimates of logistic regression model.

ICU - intensive care unit; BIC - Bayesian Information Criterion; Min - minimum; AIC - Akaike information criterion.

**Figure 3:**
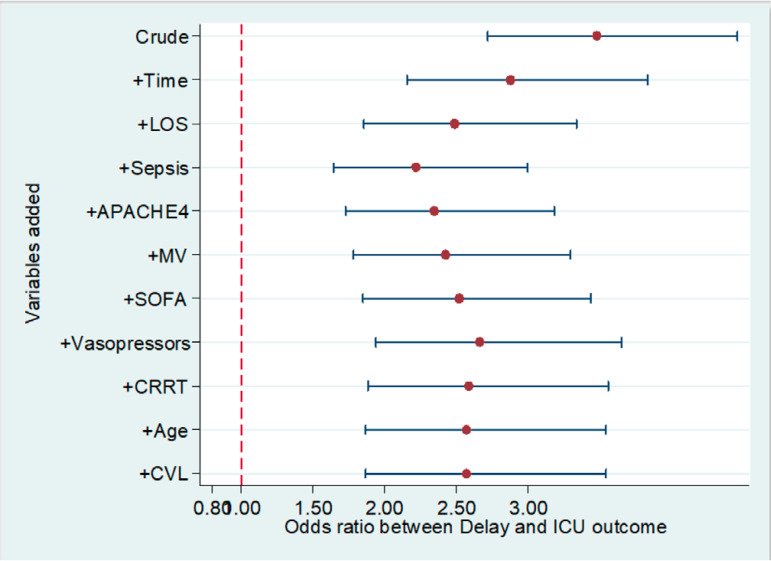
Variability in estimates of the logistic regression model.

LOS - length of stay; APACHE - *Acute Physiology and Chronic Health Evaluation*; MV - mechanical ventilation; SOFA - *Sequential Organ failure Assessment*; CRRT - continuous renal replacement therapy; CVL - central venous line; ICU - intensive care unit.

The same LR model was applied to the propensity score-matched groups, and the independent association between a delayed admission and ICU outcome persisted (OR = 2.5; 95%CI 1.8 - 3.7; p < 0.001) (Table 7S - Appendix). There was no interaction between a delay and any of the variables in the model (Figure 2S - Appendix).

### Shortest period of emergency department stay associated with intensive care unit outcomes

**Figure 4:**
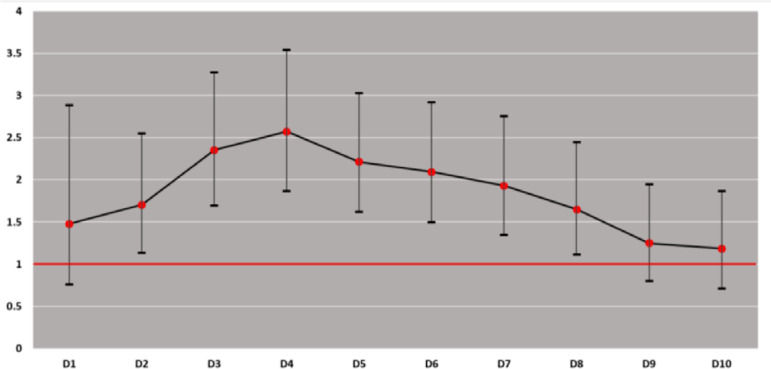
Association of delay with intensive care unit outcome by different stratifications.

We repeated the final LR model several times, changing the delay definition each time from 1 hour to 10 hours. Figure 4 indicates that the association starts after a delay of 2 hours and then disappears after 9 hours. The strongest association, however, was observed after a delay period of 4 hours.

### Subgroups and potential confounders

**Figure 5:**
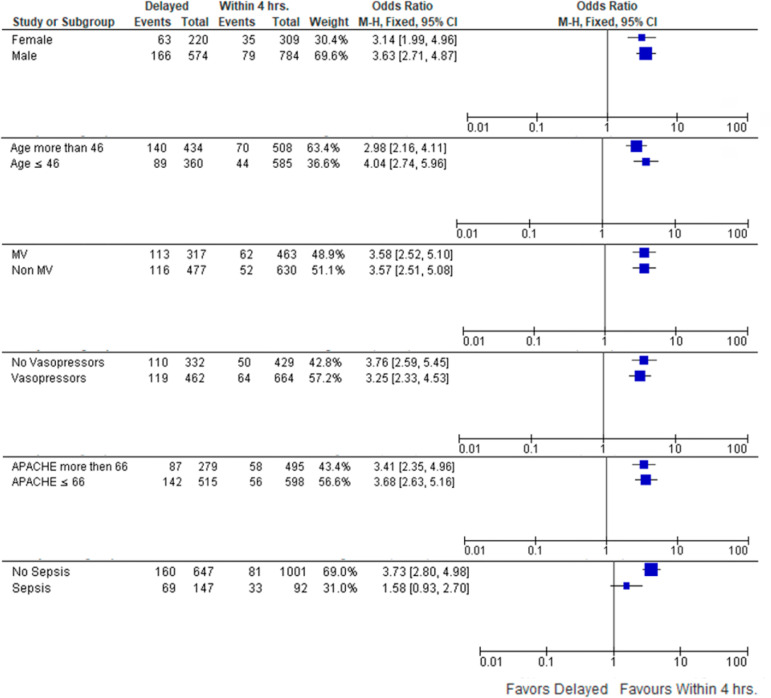
Subgroups' analysis.

The beneficial effect of admission within 4 hours on ICU mortality persisted across the strata in 5 of the predefined subgroups: age (above or below the cohort median of 46 years), sex, mechanical ventilation, vasopressors, and APACHE score 4 (above or below the cohort median of 66). Patients without sepsis benefited significantly from early admission, while for patients with sepsis, there were no statistically significant benefits (Figure 5). These results were consistent with our confounding effect analysis, where the only p-value < 0.1 of Tarone's test was for stratification by sepsis, indicating a confounding effect (Table 8S - Appendix). In view of this potential confounding effect of sepsis, the LR model was repeated excluding sepsis (Table 9S - Appendix) and it still resulted in a significant independent association of a delayed admission with ICU mortality (OR = 3, 95%CI 2.2 - 4.1; p < 0.001).

M-H - Mantel Haenszel; 95%CI - 95% confidence interval; MV - mechanical ventilation; APACHE - Acute Physiology and Chronic Health Evaluation.

## DISCUSSION

In this retrospective study of close to 2000 patients, over half were admitted within the widely accepted four-hour target. Forty-two percent were in the ED for more than four hours before admission. Those admitted within four hours were younger but sicker (higher APACHE IV and SOFA scores). This is mirrored by other studies^(1,7,8)^ and may reflect selection bias when prioritizing ICU admission. Patients with medical diagnoses, sepsis, and those requiring CRRT were more commonly delayed. While speculative, this could be due to the complex nature of their condition and/or the need for time-consuming diagnostic workups and/or the initiation of therapy in the ED.^(3,5)^

Our results show that admissions that occurred over four hours were associated with clinically important detriments. These included a longer ICU LOS and higher ICU and in-hospital mortality than patients admitted within 4 hours. These findings also mirror other studies,^(1,3,6,20)^ even those with different definitions of delay and types of patients. Taken together, this body of work supports the hypothesis that delayed patients are denied timely interventions and/or the benefit of ICU expertise, intensive monitoring, and high-intensity nursing.^(7)^ Our results are supported by a PSM that only found imbalances between groups in the distribution of sepsis; however, statistically significant differences in ICU LOS, ICU and hospital mortality persisted in favor of timely admission.

These results are not always consistent across similar studies. For example, Al-Qahtani et al.^(1)^ demonstrated lower hospital mortality and ICU LOS, though not ICU mortality, for patients admitted within six hours. Conversely, there was no difference in hospital mortality and ICU LOS between sepsis patients admitted within six hours in a study by Agustin et al.^(9)^ In one study that defined a delay as being at least eight hours, the ICU mortality was higher for the nondelayed group.^(7)^ The inconsistency of reported results likely results from differences in both inclusion criteria and definitions of what constitutes a clinically important delay.

After adjusting for demographic and clinical variables, admission after more than four hours was independently associated with a 160% increase in the odds of ICU mortality in a well-fitted model that withstood two sensitivity tests and remained significant after PSM. Several studies support our findings. Chalfin et al.^(3)^ demonstrated a lower adjusted odds of hospital survival after a delayed admission of more than six hours. In a study of nontrauma ventilated patients,^(21)^ a delayed admission of more than four hours was significantly associated with ventilation mortality within 21 days (OR 1.41; 95%CI 1.05 - 1.89). With a cutoff value of a delayed admission of five hours, García-Gigorro et al.^(6)^ demonstrated an OR of 2.5 (95%CI 1.3 - 4.7) for in-hospital mortality. In contrast, other studies found no association. Al-Qahtani et al.^(1)^ showed an insignificant adjusted OR for ICU mortality with a delay of more than six hours. Similar results were reported for in-hospital mortality of sepsis patients with a six-hour delay,^(9)^ and in the study by O'Callaghan et al.,^(22)^ the odds of ICU mortality was insignificant with a delayed admission over three hours. A Dutch study^(11)^ even reported a negative correlation between a delay of more than 3.7 hours and hospital mortality (OR = 0.82; 95%CI 0.72 - 0.92) compared to less than 1.2 hours.

The wide variation in results cannot be solely based on differences in delay demarcation or inclusion criteria, since studies that demonstrated a significant association also have these differences among them. Accordingly, a reasonable explanation in view of the hypothesis of worse outcomes due to delayed ICU management and expertise would be that different outcomes may be explained by how closely the care in the ED resembles that of an ICU. If the ED is well equipped and staffed to provide care for the critically ill, then we may not expect worse outcomes for delayed patients, but since most EDs are not,^(11,23,24)^ then a delayed ICU admission may negatively impact patients' outcomes. This hypothesis is supported by several studies. In a large-scale Canadian study,^(25)^ ED crowding was associated with an ED LOS of more than 6 hours (OR 1.19; 95%CI 1.19 - 1.19) but was also associated with the same 90-day mortality (OR 1.01; 95%CI 1.01 - 1.01). Chan et al.^(26)^ reported longer waiting times and care times with fewer nursing staffing in the ED. In a Turkish study,^(27)^ the nursing ratio in the ED was significantly associated with adverse events.

Association is not causation; however, our results fulfill most of the Bradford Hill criteria of a causal relationship between exposure and outcome.^(28)^ Biological grading is also shown in our results, since the time spent in the ED (as a continuous variable in minutes) was significantly associated with a 3.5% increase in the odds of ICU mortality for each hour spent in the ED. The criterion of strength is shown by a considerably high OR with a highly significant p-value.

The association of a delay with worse outcomes in our study emerged after a delay of two hours; however, the strongest association was after a delay of four hours. For this reason, we believe that the best timing of admission would be within 4 hours but does not necessarily have to be less than 3 hours, since we did not study the period between 3 and 4 hours delay, and the benefit of admission within three ours was not so large statistically. On the other hand, we cannot consider admission after 9 hours to be futile, but we believe that the benefit of early admission would be lost, and other prognostic factors (such as case severity) would become the main determinants of the outcome.

In subgroup analyses, sepsis appeared to confound the impact of delayed admission on ICU mortality. A similar result was shown in the subgroup of sepsis patients in the study by Chalfin et al.,^(3)^ where delayed sepsis patients had significantly higher ICU mortality (27.8% *versus* 20.4%, p = 0.06) than nondelayed patients. Sepsis fulfilled all criteria of a potential confounder,^(29)^ being associated with a delay while being unevenly distributed in both groups even after PSM. It was independently associated with ICU mortality and was not in the causal pathway between delay and outcome. Sepsis is known to be associated with a high mortality,^(30)^ and similarly, it may be associated with delayed admission in view of the numerous therapeutic interventions involved in its management (central line insertion, initiation of vasopressors, CRRT).^(9)^ Removal of sepsis from the LR still resulted in a significant association between delayed admission and ICU mortality.

Our study suffers from numerous limitations. The first is the limitation inherent within a retrospective observational design. Second, it is a single-center study, reflecting practice in only one institute. Third, a description of the ED profile (number and specialties of the physicians, nurses, patients etc.) at the time of the study was not available to us, and we could not retrieve it from the ED. Although an analysis of the correlation of such factors with a delay would have been a point of strength in our study, we acknowledge that a lack of such an analysis is a limitation of our study and that this may be the basis of further research. Last, we did not discriminate between patients with and without a restrictive order (do not resuscitate) with regard to ICU and hospital mortality.

## CONCLUSION

Delayed admission to the intensive care unit from the emergency department is an independent risk factor for increased intensive care unit mortality. A delay period of four or more hours is associated with worse outcomes.

## Figures and Tables

**Figure 1S:**
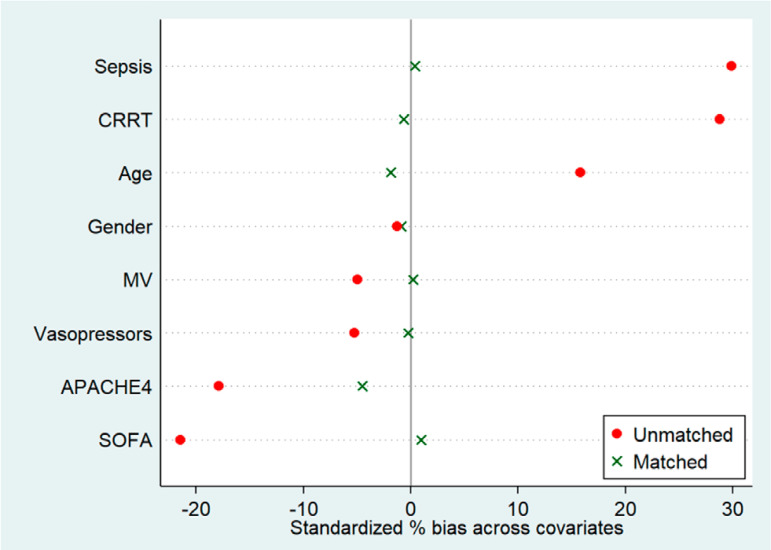
Variables percentage of bias reduction after matching.

CRRT - continuous renal replacement therapy; MV - mechanical ventilation; APACHE - Acute Physiology and Chronic Health Evaluation; SOFA - Sequential Organ failure Assessment.

**Figure 2S:**
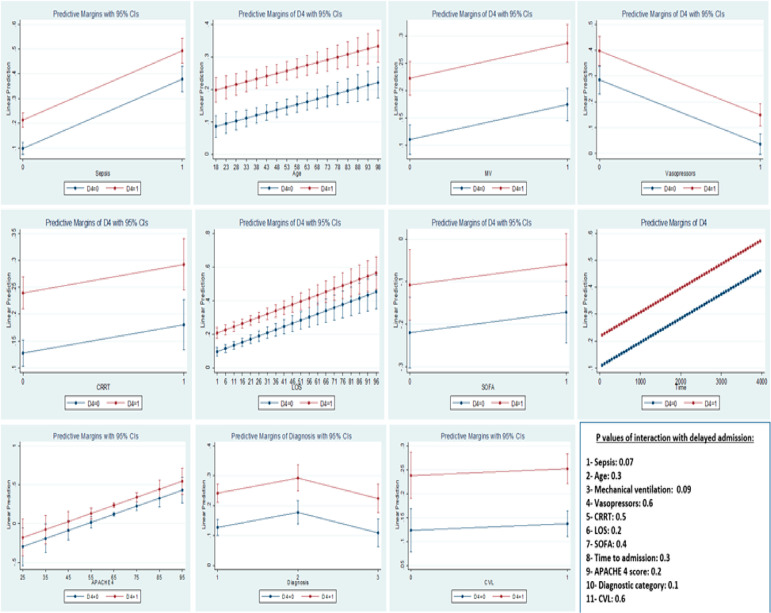
Interaction plots between delay and other variables in logistic regression model.

95%IC - 95% of confidence interval; MV - mechanical ventilation; CRRT - continuous renal replacement therapy; LOS - length of stay; SOFA - Sequential Organ failure Assessment; APACHE - Acute Physiology and Chronic Health Evaluation; CVL - central venous line.

## APPENDIX 1 Content

1. Table 1S: Recorded variables and missing data.
2. Table 2S: Diagnostic categories and top five diagnoses.
3. Table 3S: Matched Groups: demographics and clinical characteristics.
4. Table 4S: Secondary outcomes of matched groups.
5. Table 5S: Variable Inflation Factors of multi-variate logistic regression model.
6. Table 6S: Linearity of independent variables and Log odds (Box-Tidwell test).
7. Table 7S: Logistic regression of matched groups.
8. Table 8S: Tarone's test of homogeneity of Crude and Stratified odds ratio between delayed admission and intensive care unit mortality.
9. Table 9S: Logistic regression model excluding sepsis.
10. Figure 1S: Variables percentage of bias reduction after matching.
11. Figure 2S: Interaction plots between Delay and other variables in logistic regression model.

**Table 1S:** Recorded variables and missing data

Data: 1,887 patients	n (%)
Age (years)	0 (0)
Gender (male - female)	0 (0)
MV (yes - no)	0 (0)
Time to admit (minutes)	0 (0)
ICU length of stay (days)	0 (0)
ICU outcome (alive - dead)	0 (0)
Hospital outcome (alive - dead)	0 (0)
Diagnostic category (medical - surgical - trauma)	0 (0)
APACHE IV	0 (0)
SOFA	19 (1)
CVL (yes - no)	0 (0)
CRRT in ER (yes - no)	0 (0)
Vasopressors in ED	0 (0)
MEWS in ER	159 (8.4)

MV - mechanical ventilation; ICU - intensive care unit; APACHE - Acute Physiology and Chronic Health Evaluation; SOFA - Sequential Organ failure Assessment; CVL - central venous line; CRRT - continuous renal replacement therapy; ED - emergency department; MEWS - modified early warning score. Results expressed as n (%).

**Table 2S:** Diagnostic categories and top 5 diagnoses

Medical (n = 1,233)	n (%)
Infection related respiratory (eg. CAP - H1N1)	516 (42)
Non-traumatic neurological (eg. ischemic stroke - spontaneous ICH)	312 (25)
Septic shock/sepsis	220 (18)
COPD	107 (8)
Endocrinal and electrolyte disturbances (eg. diabetic keto-acidosis)	47 (4)
**Surgical (n = 356)**	
Upper/lower gastrointestinal tract bleeding	114 (32)
Gastrointestinal perforation	102 (29)
Intestinal obstruction	64 (18)
Hepato-biliary	47 (13)
Sepsis related surgical (diabetic foot - surgical site infection)	19 (5)
**Trauma (n = 298)**	
Road traffic accident polytrauma	163 (55)
Isolated traumatic head injury	63 (21)
Orthopedic related trauma	30 (10)
Abdominal trauma (internal hemorrhage - retroperitoneal hematoma)	24 (8)
Vascular injuries	10 (3)

CAP - community acquired pneumonia; ICH - intracerebral hemorrhage; COPD - chronic obstructive pulmonary disease.

**Table 3S:** Matched Groups: demographics and clinical characteristics

	Delayed (n = 794)	Within time (n = 794)	p value
Age	49.9 ± 19.9	50.3 ± 19.6	0.7
Males	574 (72.3)	571 (71.9)	0.9
MV	317 (39.9)	316 (39.8)	0.99
Diagnosis			
Medical	558 (70.3)	556 (70)	0.9
Surgical	110 (13.9)	111 (14)	0.9
Trauma	126 (15.8)	127 (16)	0.9
CVL	577 (72.7)	575 (72.4)	0.9
Vasopressors	462 (58.2)	463 (58.3)	0.9
CRRT	191 (24.1)	193 (24.3)	0.9
Sepsis	147 (18.5)	132 (12.1)	< 0.001
APACHE IV	65.9 ± 2.8	66.1 ± 3	0.2
SOFA	7.1 ± 2.9	7.03 ± 2.9	0.6
MEWS	2.4 ± 2	2.6 ± 2.3	0.06
Time to admission	625.2 ± 485.4	162.4 ± 88.8	< 0.001

MV - mechanical ventilation; CVL - central venous line; CRRT - continuous renal replacement therapy; APACHE - Acute Physiology and Chronic Health Evaluation; SOFA - Sequential Organ failure Assessment; MEWS - Modified Early Warning Score. Results expressed as n (%) or mean ± standard deviation.

**Table 4S:** Secondary outcomes of matched groups

	Dealyed (n = 794 )	Within time (n = 794)	p value
ICU length of stay	15.2 ± 17.1	12 ± 14.1	< 0.001
ICU mortality	229 (28.8)	104 (13.1)	< 0.001
Hospital mortality	279 (35.1)	151 (19)	< 0.001

ICU - intensive care unit; LOS - length of stay. Results expressed as n (%) or mean ± standard deviation.

**Table 5S:** Variable Inflation Factors of multi-variate logistic regression model

Variable	Variance inflation factor
Vasopressors	3.81
SOFA	3.18
Central venous line	2.34
CRRT	1.38
Time to admission	1.31
Sepsis	1.26
APACHE IV	1.24
Diagnosis	1.22
Age	1.14
Delay admission	1.10
Length of stay	1.09
Mechanical ventilation	1.07
Mean VIF	1.68

SOFA - *Sequential Organ failure Assessment*; CRRT - continuous renal replacement therapy; APACHE - *Acute Physiology and Chronic Health Evaluation*; VIF - *variance inflation factor.*

**Table 6S:** Linearity of independent variables and Log odds (Box-Tidwell test)

Continuous variable	Box-Tidwell p value
Age	0.179
Time to admission	0.248
Length of stay	0.212
APACHE IV	0.092
SOFA	0.142

APACHE - Acute Physiology and Chronic Health Evaluation; SOFA - Sequential Organ failure Assessment.

**Table 7S:** Logistic regression of matched groups

Variable	Multivariate logistic regression		
	OR	95%CI	p valor
Age	1.01	1.005 - 1.02	0.001
MV	1.8	1.3 - 2.4	< 0.001
CRRT	2.3	1.5 - 3.7	< 0.001
Vasopressors	0.06	0.02 - 0.14	< 0.001
Central venous line	1.2	0.7 - 1.9	0.5
Diagnosis			
Medical	Reference		
Surgical	1.1	0.8 - 1.7	0.5
Trauma	0.6	0.3 - 0.9	0.02
APACHE IV	1.05	0.99 - 1.1	0.08
SOFA	1.6	1.4 - 1.8	< 0.001
Sepsis	3.7	2.9 - 4.5	< 0.001
Time to admission	1.0004	1.0001 - 1.0008	0.008
ICU length of stay	1.02	1.01 - 1.03	< 0.001
Delay admission	2.5	1.8 - 3.7	< 0.001

OR - odds ratio; 95%CI - 95% of confidence interval; MV - mechanical ventilation; CRRT - continuous renal replacement therapy; APACHE - Acute Physiology and Chronic Health Evaluation; SOFA - Sequential Organ failure Assessment; ICU - intensive care unit.

**Table 8S:** Tarone's test of homogeneity of crude and stratified odds ratio between delayed admission and intensive care unit mortality

Estimate	Mantel-Haenszel combined OR	95%CI	Tarone's p value
Crude	3.480686	2.700016 - 4.498318	
Gender	3.481862	2.718713 - 4.459228	0.6017
Age (above/below median)	3.367081	2.626465 - 4.316538	0.2388
MV	3.57704	2.787529 - 4.590164	0.9904
Vasopressors	3.467953	2.70709 - 4.442666	0.5720
APACHE IV (above/below median)	3.567202	2.778645 - 4.579545	0.7659
Sepsis	3.064409	2.379832 - 3.945909	0.0055

OR - odds ratio; 95%CI - 95% of confidence interval; MV - mechanical ventilation; APACHE - Acute Physiology and Chronic Health Evaluation.

**Table 9S:** Logistic regression model excluding sepsis

Variable	Multi-variate model		
	OR	95%CI	p value
Age	1.01	1.007 - 1.02	< 0.001
MV	1.8	1.4 - 2.4	< 0.001
CRRT	1.7	1.1 - 2.7	< 0.001
Vasopressors	0.15	0.07 - 0.3	0.06
Central venous line	0.9	0.6 - 1.4	0.7
Diagnosis			
Medical	Reference		
Surgical	1.2	0.9 - 1.7	0.2
Trauma	0.6	0.4 - 0.9	0.01
APACHE IV	1.06	1.01 - 1.1	0.02
SOFA	1.4	1.3 - 1.5	< 0.001
Time to admission	1.0005	1.0001 - 1.001	0.006
ICU length of stay	1.03	1.02 - 1.03	< 0.001
Delay admission	3	2.2 - 4.1	< 0.001

OR - odds ratio; 95%CI - 95% of confidence interval; MV - mechanical ventilation; CRRT - continuous renal replacement therapy; APACHE - Acute Physiology and Chronic Health Evaluation; SOFA - Sequential Organ failure Assessment; ICU - intensive care unit.
